# Incidence of complications among *in vitro* fertilization pregnancies

**DOI:** 10.25122/jml-2023-0048

**Published:** 2023-03

**Authors:** Raluca Tocariu, Daniela Stan, Raluca Florina Mitroi, Daniela Elena Căldăraru, Alexandru Dinulescu, Claudia Elena Dobre, Elvira Brătilă

**Affiliations:** 1 Department of Obstetrics and Gynecology, Carol Davila University of Medicine and Pharmacy, Bucharest, Romania; 2 Department of Obstetrics and Gynecology, Clinical Hospital of Obstetrics and Gynecology Prof. Dr. Panait Sîrbu, Bucharest, Romania; 3 Department of General Nursing, Carol Davila University of Medicine and Pharmacy, Bucharest, Romania; 4 Department of Pediatrics, Grigore Alexandrescu Emergency Hospital for Children, Bucharest, Romania

**Keywords:** infertility, in vitro fertilization, pregnancy complications, perinatal complications

## Abstract

The use of assisted reproductive technology has increased in Romania in the past several years. Although most of these pregnancies are uncomplicated, in vitro fertilization is associated with an increased risk for adverse perinatal outcomes primarily caused by the increased risks of prematurity, gestational diabetes mellitus, and hypertensive disorders. Infertility can be caused by a variety of factors, including both male and female factors, and in some cases, the cause remains unknown. In our clinic, the etiology of infertility was known in most cases and was equally distributed between male and female factors. Women with gestational hypertension were significantly older. Patients with twin pregnancies were significantly younger than those with a single pregnancy. The prevalence of preterm newborns was 2.5 times higher than the global prevalence for prematurity.

## INTRODUCTION

Infertility is a medical condition defined as the inability to achieve a clinical pregnancy within 12 months of regular sexual intercourse without contraceptive measures. This condition affects approximately 186 million individuals worldwide, with an identifiable cause in most cases estimated to be around 85% of affected couples. Advanced maternal age has been identified as the most significant negative predictive factor for pregnancy, with a decline observed around 25-30 years [1,2].

In vitro fertilization (IVF) was first used in England in 1978 and has become widely available with increasing demand. Initially, IVF was used for women with bilateral tubal occlusion, but its indications were extended in recent years to include other causes of infertility, including unexplained causes. The success of the procedure is influenced by various predictive factors, such as women’s age, the basal value of FSH, the number and quality of embryos used for the transfer, and causes of infertility. In the United States, 1-3 % of all births each year are the result of IVF [3-5].

The first successful IVF birth in Romania was reported on February 6, 1996, approximately 18 years later than the first IVF birth in the world. In 2012, there were 26 clinics providing assisted reproductive services in Romania [6]. However, due to the lack of clear statistics on assisted reproductive procedures in Romania, it is currently unclear how many clinics perform IVF and the annual number of IVF newborns. In 2011, the Romanian government introduced a national sponsorship program to assist couples struggling with infertility, providing financial aid to cover a part of the IVF procedure [6]. In 2022, the Romanian government continued to provide financial aid for the IVF procedure, with a subsidy of 15 000 Romanian lei (approximately 3000 €)[7].

IVF is associated with an increased risk of complications like hypertension, gestational diabetes mellitus, prematurity, and other perinatal complications [8,9]. Hypertension-related complications can affect the mother and the fetus and are observed in 2-3% of pregnancies. These complications are divided into chronic hypertension, gestational hypertension, and preeclampsia. Chronic hypertension is defined as high blood pressure before the pregnancy, diagnosed in the first 20 weeks of gestation, or a high blood pressure that does not resolve 12 weeks postpartum. Gestational hypertension (formerly known as pregnancy-induced hypertension) is defined as high blood pressure after 20 weeks of pregnancy with normal blood pressure before the pregnancy and no signs of preeclampsia or proteinuria. Preeclampsia, which has an unknown etiology, is a disease that affects multiple organs and is characterized by high blood pressure and proteinuria after 20 weeks of pregnancy. In vitro fertilization is associated with an increased risk of hypertensive disorders during pregnancy, especially in frozen embryo transfer, compared with natural conception or fresh embryo transfer. Although the perinatal and obstetric outcomes in frozen embryo transfer were better than those in fresh embryo transfer, the risk for these complications was approximately 2 times higher in frozen embryo transfer than natural conception and 1.5 times higher than fresh embryo transfer [10–13].

The prevalence of gestational diabetes mellitus (GDM) varies by region and diagnostic criteria, with global prevalence reported as 7-25% and approximately 14.7% in some reports. Gestational diabetes mellitus is defined as glucose intolerance that is first discovered during gestation. Many factors, including obesity, multiple pregnancies, and in vitro fertilization, influence the risk of developing GDM. GDM is associated with an increased risk of preeclampsia and prematurity [14–17].

Prematurity or preterm birth is defined as a newborn with a gestational age of fewer than 37 weeks. Subcategories of prematurity include moderately or late preterm (33-36 weeks), very preterm (28-32 weeks), and extremely preterm (less than 28 weeks). The prevalence of prematurity is estimated to be around 5-18%, according to the World Health Organization (WHO). Although the pathophysiology of prematurity is largely unknown, there are identified predisposing factors, including assisted reproduction. Limiting the number of implanted embryos has improved the prematurity rate in IVF pregnancies [18–20].

The objective of this study was to analyze IVF pregnancies and to identify the major complications and risk factors associated with this procedure.

## MATERIAL AND METHODS

This observational retrospective longitudinal study included 155 female patients who underwent IVF at the Clinical Hospital of Obstetrics and Gynecology Prof. Dr. Panait Sîrbu, in Bucharest, between January 2021-December 2022. The study was conducted according to the protocol approved by the Clinical Hospital of Obstetrics and Gynecology ethics committee Prof. Dr. Panait Sîrbu (nr.33/3.11.2022). The inclusion criteria were women who had infertility and underwent an embryo transfer procedure with their own oocyte or egg donation in the clinic. Patients with incomplete chart data were excluded from the study.

We collected data on age, number of embryos transferred, maturity of the embryos, infertility etiology, and developed pathologies during the pregnancy. The data were collected from the electronic register of the hospital and the patients’ charts.

The sample size was calculated using the following formula:


$$n=\frac{z^{2}*\hat{P}\left(1-\hat{P}\right)}{\varepsilon^{2}}$$

n is the sample size; z is the z-score; p̂ is the population proportion; ε is the margin of error (confidence interval).

The sample size was calculated with a confidence interval of 95% and a margin error of ±2.69%. No control group was used in the study. To minimize bias, simple random sampling was used for data collection. Data was collected in Microsoft Excel and analyzed using IBM SPSS version 26. The Shapiro-Wilk test was used to assess the distribution of quantitative variables, which were reported as averages with standard deviations or medians with interquartile ranges according to their distribution. Quantitative variables were compared between independent groups using Mann-Whitney U tests or independent sample t-test depending on their distribution. Fisher's exact test was used to determine if there were nonrandom associations between two categorical variables.

## RESULTS

**Figure 1:**
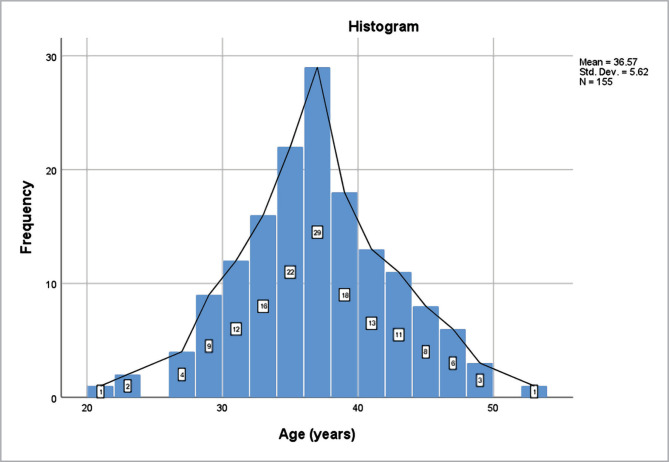
Age distribution of patients included in the study.

For analysis, 155 patients with infertility were selected. The patients were normally distributed by age (Shapiro-Wilk test p=0.299), with a mean of 36,57 years (±5.62) (Figure 1). The age range of the patients varied from 21 to 53 years old.

**Figure 2:**
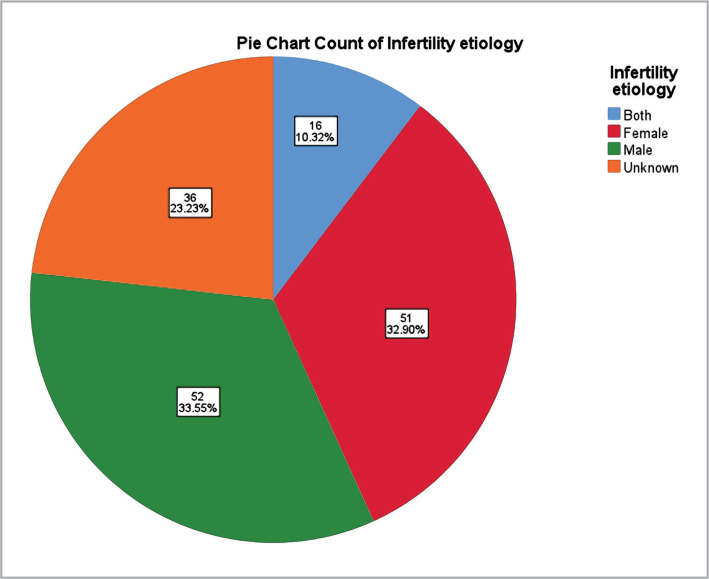
Infertility by etiology.

The majority of infertility cases in the studied sample were attributed to male factors (33.5%), followed by female factors (32.9%), unknown causes (23.2%), and combined factors (10.3%), as shown in Figure 2.

**Figure 3:**
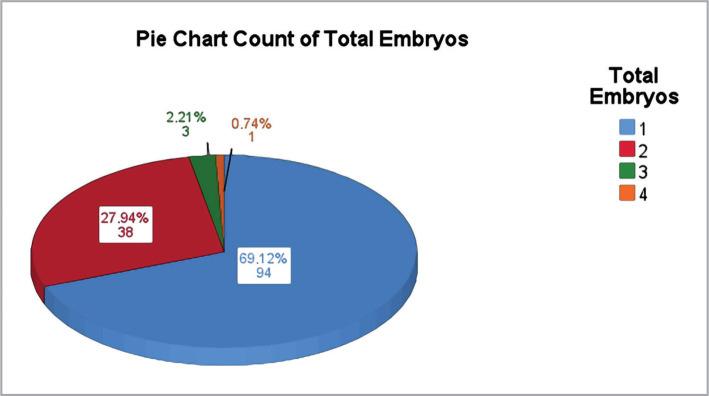
Total embryos used in each embryo transfer.

Most patients (87.7%) underwent an embryo transfer procedure using their own oocyte, while 9% used egg donation and 3.2% used artificial insemination. 94 patients (69.12%) used the transfer of one embryo per cycle, while 38 (27.94%) transferred 2 embryos per cycle (Figure 3).

**Figure 4:**
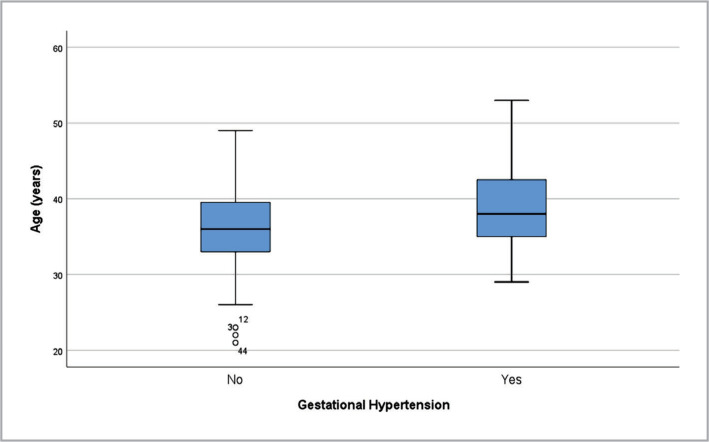
Gestational hypertension by age.

Out of 155 patients, 121 (78.1%) had embryo transfer with day 5 embryos, 38 patients (24.5%) developed gestational diabetes, and 28 developed gestational hypertension (18.1%). The mean age of patients with gestational diabetes was 37.87 years (±5.23*) compared to 36.15 (±5.69*), although the difference was not statistically significant (p=0.089**). However, the group with gestational hypertension was significantly older, with a mean age of 38.86 years (±5.84*) compared to 36,06 years (±5,46*) (p=0.026**) (Figure 4).

Pregnancy termination and birth occurred in 137 patients. Of these, 104 pregnancies (67.1%) were single, and 33 (24.1%) were twin pregnancies.

**Figure 5:**
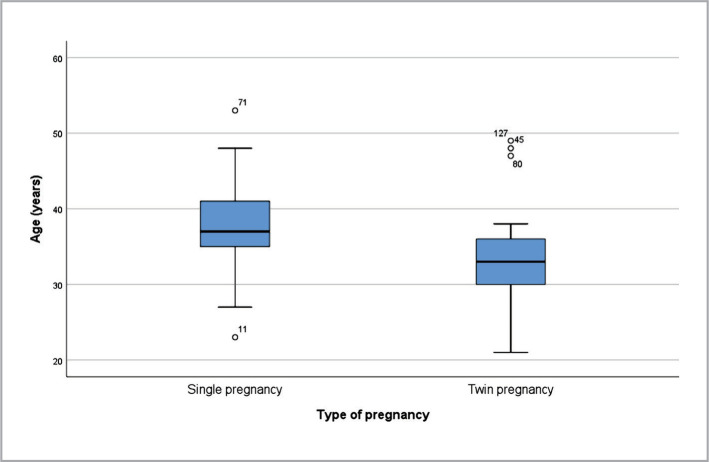
Type of pregnancy by age.

The age distribution of mothers differed significantly between the two types of pregnancies, with a non-normal distribution as determined by the Shapiro-Wilk test (p<0.05*). Mothers of twin pregnancies were found to be significantly younger than those of single pregnancies, with a median age of 33 years (interquartile range [IQR]: 30-36.5**) compared to 37 years (IQR: 35-41**) for mothers with single pregnancies (p<0.001***) (Figure 5).

Table 1 shows the distribution of patients according to the IFV technique and the occurrence of multiple pregnancies. Significant differences were observed between groups using Fisher’s Exact Test (p<0.001). Post-hoc Z-tests with Bonferroni correction revealed that embryo transfer with 2 embryos was significantly associated with a higher twin pregnancy rate than a transfer with 1 embryo (63.6% vs. 6.1%). No significant associations between the other techniques and multiple pregnancies were found.

**Table 1:** Distribution of twin pregnancies according to the IVF technique.

IVF technique/type of pregnancy	Single pregnancy		Twin pregnancy		Fisher’s exact test, P-value
	N	%	N	%	
Embryo transfer with 1 embryo	**77**	**93.9%**	**5**	**6.1%**	**<0.001**
Embryo transfer with 2 embryos	**12**	**36.4%**	**21**	**63.6%**	
Embryo transfer with 3 embryos	2	66.7%	1	33.3%	
Embryo transfer with 4 embryos	1	100%	0	0%	
Artificial insemination	2	50%	2	50%	
Egg donation	10	71.4%	4	28.6%	-

No significant correlation was found between gestational diabetes or gestational hypertension and the type of pregnancy (single or twin) as determined by Fisher's exact test (p>0.05*).

Of 131 pregnancies, 34 (25.9%) resulted in preterm newborns. The age distribution of mothers based on the gestational age of the newborn had a normal distribution (Shapiro-Wilk test, p>0.05*). Women with preterm newborns were younger than the ones with term newborns, 35.5 (±6.95**) years vs. 37.18 (±5.23**) years, but this was not statistically significant (Independent Sample T-Test, p=0.144***).

There was no significant correlation between gestational diabetes or gestational hypertension and gestational age (Fisher's exact test, p>0.05*). Furthermore, there was no correlation between the infertility etiology and the gestational age (Fisher's exact test, p=0.646*). Nine out of 34 preterm newborns (26.4%) presented acute respiratory distress syndrome (ARDS).

## DISCUSSION

In this study, we aimed to analyze IVF pregnancies and associated complications and risk factors. Infertility has several etiologies, both male and female, and in some cases, the cause remains unknown. The distribution of male/female factors varies in the literature, with a female predominance reported more frequently. However, in our study, male and female infertility had a similar prevalence (33.5% and 32.9%), followed by unknown causes (23.2 %) and combined factors as the least common (10.3%). These findings are similar to those reported by Masoumi et al. and to the unknown and combined causes described by Deshpande and Gupta [21–24].

The majority of those who underwent embryo transfer, 121 (89%), used day 5 embryos. While there is uncertainty about whether there is a difference in live birth rates between day 3 and day 5 embryos, a study by Wang et al. (2021) found a better live birth rate in day 5 embryos [25–27]. Furthermore, a correlation was found between the number of embryos used in embryo transfer and the multiple pregnancy rate, which was also observed in our study. Embryo transfer with 2 embryos was significantly more associated with twin pregnancy than embryo transfer with 1 embryo (63.6% vs. 6.1%) (p<0.001). In our study, the number of embryos used in each pregnancy was in accordance with the guidelines established by The American Society for Reproductive Medicine, which promote the transfer of fewer embryos to reduce the number of multiple pregnancies. It is worth noting that the pregnancy rate in one embryo transfer was approximately 40%, according to previous research [28–30].

Complications of pregnancies include gestational hypertension and GDM. According to the literature, the prevalence of these complications is higher in IVF than in spontaneous pregnancies, with rates of 4.5% vs. 3.6 % for gestational hypertension and 6.6% vs. 4.4% for GDM [11,31]. However, in our study, the prevalence of these complications was much higher, with rates of 18.1% for gestational hypertension and 24.5% for GDM. In line with previous research, the gestational hypertension group in our study was significantly older (p=0,026) [32,33].

Although advanced maternal age is a known risk factor for multiple pregnancy, this correlation may also be attributed to the increased use of IVF in this population, as IVF is an independent risk factor for multiple pregnancy. According to Tough et al., the prevalence of multiple pregnancy in IFV was higher in women under 30, although this finding was not statistically significant [34–36]. In our study, women with twin pregnancies were significantly younger than single pregnancies (p<0.001).

The global prevalence of preterm birth was around 11.1% in 2010, with IVF being a risk factor for premature birth. The prevalence of preterm birth in our study was much higher than the global prevalence, with a quarter (25.9%) of the newborns being preterm. Advanced maternal age is recorded in the literature as a risk factor for very premature birth, but in our study, there was no correlation between the age of mothers and prematurity [37–40]

Our study has several limitations that should be considered when interpreting the results. Firstly, we did not have data about essential factors such as body mass index or other potential confounding variables that could influence the occurrence of pregnancy complications. Additionally, we lacked data on the total embryos used in each embryo transfer, day of the embryos, the type of pregnancy (single/multiple), or gestational age in all 155 patients included in the study. These limitations may affect the generalizability of our findings and warrant further investigation in future studies.

## CONCLUSION

The mean age of women who underwent IFV was 36.57 (±5.62) years. In most cases, the etiology was known, and male and female factors were equally distributed as etiologies of infertility. The use of fewer embryos in the transfer was consistent with the guidelines recommended by the American Society for Reproductive Medicine. The number of embryos used was correlated with the incidence of multiple pregnancies. 24.5% of participants had gestational diabetes, and 18.1% had gestational hypertension. Gestational hypertension was more common in older women. 24.1% of pregnancies were twin pregnancies, and women with twin pregnancies were significantly younger than those with single pregnancies. 25.9% of participants had preterm newborns, 2.5 times higher than the global prevalence for prematurity, and there was no statistically significant difference between the age of the mothers with preterm newborns.

## Data Availability

The data is available from the corresponding author upon reasonable request.
